# Fitness Benefits of Mate Choice for Compatibility in a Socially Monogamous Species

**DOI:** 10.1371/journal.pbio.1002248

**Published:** 2015-09-14

**Authors:** Malika Ihle, Bart Kempenaers, Wolfgang Forstmeier

**Affiliations:** Department of Behavioral Ecology and Evolutionary Genetics, Max Planck Institute for Ornithology, Seewiesen, Germany; University of New South Wales, AUSTRALIA

## Abstract

Research on mate choice has primarily focused on preferences for quality indicators, assuming that all individuals show consensus about who is the most attractive. However, in some species, mating preferences seem largely individual-specific, suggesting that they might target genetic or behavioral compatibility. Few studies have quantified the fitness consequences of allowing versus preventing such idiosyncratic mate choice. Here, we report on an experiment that controls for variation in overall partner quality and show that zebra finch (*Taeniopygia guttata*) pairs that resulted from free mate choice achieved a 37% higher reproductive success than pairs that were forced to mate. Cross-fostering of freshly laid eggs showed that embryo mortality (before hatching) primarily depended on the identity of the genetic parents, whereas offspring mortality during the rearing period depended on foster-parent identity. Therefore, preventing mate choice should lead to an increase in embryo mortality if mate choice targets genetic compatibility (for embryo viability), and to an increase in offspring mortality if mate choice targets behavioral compatibility (for better rearing). We found that pairs from both treatments showed equal rates of embryo mortality, but chosen pairs were better at raising offspring. These results thus support the behavioral, but not the genetic, compatibility hypothesis. Further exploratory analyses reveal several differences in behavior and fitness components between “free-choice” and “forced” pairs.

## Abstract 2

A study of zebra finches shows that free mate choice leads to higher fitness than forced pairings due to increased behavioral, rather than genetic, compatibility. Read the Synopsis.

## Author Summary

The last half century has seen a tremendous interest in the study of mate choice and the evolution of traits that make individuals attractive to others. In some species, however, individuals can differ substantially in who they find attractive, and this variation has typically been interpreted as “mate choice for compatibility.” Here, we quantify the benefits of such mate choice in a socially monogamous passerine bird, the zebra finch. We found that pairs that resulted from free mate choice achieved a 37% higher reproductive success than pairs that were forced to mate with a randomly assigned individual. Forced pairs suffered from increased failure to fertilize eggs and from increased mortality of hatched offspring. In females, we observed a reduced readiness to copulate with the assigned partner, while males that were force‐paired showed reduced parental care and increased activity in courting extra‐pair females. These findings support the hypothesis that zebra finches choose mates on the basis of behavioral compatibility. In contrast, it appears that zebra finches have not evolved a mechanism that would allow them to select a partner with whom they could minimize the rate of embryo mortality. This argues against mate choice for genetic compatibility.

## Introduction

**Fig 1:**
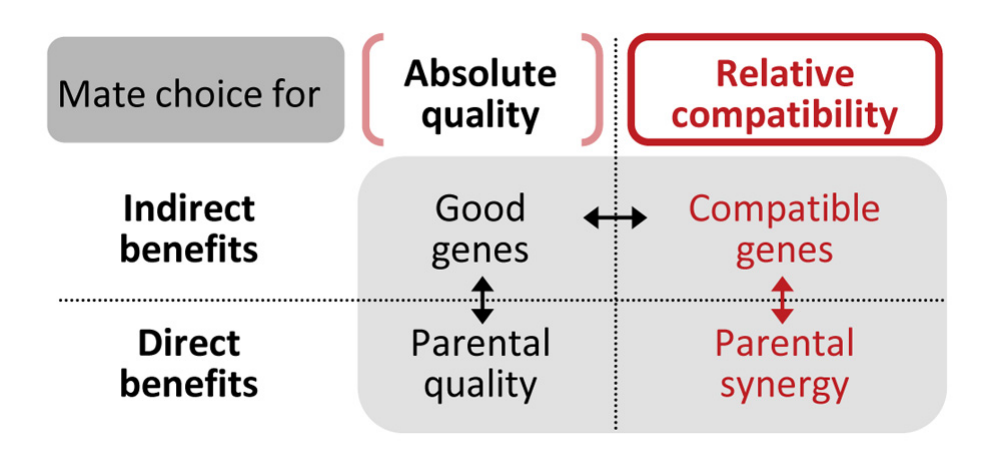
Schematic overview of four types of potential fitness benefits of mate choice. This study aims at separating direct from indirect benefits of mate choice for compatibility (red arrow), while experimentally controlling for effects of overall quality (red parentheses).

The evolution of mate choice has been the focus of much research, and many studies have attempted, with a variety of experimental approaches, to measure the fitness benefits gained by choosy individuals (e.g., [1,2–7]). Those benefits can be either *direct*, if offspring quality or quantity is increased due to the partner’s behavior (including reproductive investment), or *indirect*, if offspring quality is improved by the genetic contribution of the partner. To date, the central debate has been about (i) the relative importance of direct versus indirect fitness benefits arising from the overall quality of the chosen partner (i.e., good parent versus good genes; Fig 1, vertical black arrow) [8,9], or about (ii) the relative importance of the two types of indirect benefits (i.e., good genes versus compatible genes; Fig 1, horizontal black arrow) [10–13].

Several studies on mate choice have shown that, in some species, mating preferences can be largely specific to the individual [14–19]. Such mate preferences may function to maximize offspring viability by bringing together compatible combinations of genes (top right in Fig 1). However, the alternative hypothesis that mate choice could lead to direct benefits arising from the phenotypic (e.g., behavioral) compatibility of the two partners (bottom right in Fig 1) has received only little attention [20–26], despite suggestive evidence that the combination of both parents’ behaviors or other phenotypes can affect breeding success. Compatible partners could, for instance, be better at coordinating tasks, at sharing them or at complementing each other’s performance on various tasks [23–25,27–31], or they might simply be more effective at stimulating one another’s reproductive investment [32–34]. Mate choice for such behavioral compatibility might be especially important in species with intense bi-parental brood care and with long-lasting, monogamous pair bonds, like humans or many bird species.

Previous experiments that aimed to quantify the fitness benefits of mate choice arising from partner compatibility typically compared two categories of individuals: those paired up with their preferred partner versus those that were given a non-preferred partner [35–43], or a random partner from the population [44,45]. The problem with this approach is that the effects of individual quality and pair compatibility are confounded, because only the force-paired group includes individuals that might never have been chosen (i.e., low-quality individuals). Some studies addressed this issue by presenting evidence that the rejection of a particular mate depended on the choosing individual’s identity [46], or that non-preferred and preferred individuals did not differ in morphological traits [43]. Other studies have compared the reproductive success of a choosing individual (paired with its preferred partner) with the reproductive success of a naïve individual paired with that same (or another) preferred individual [2,47–50]. In the latter design, chosen and assigned partners are on average of equal quality. However, choosing individuals were often discarded if they did not meet a certain criterion regarding their strength of preference ([2,48,49], but see [47,50]). If choosiness is associated with an individual’s quality [17,51], the selected subset of choosing individuals might differ in quality from the random pool of naïve individuals to which they are compared. However, in two experimental studies on invertebrates, none of the above issues apply; these studies found no fitness benefit of mate choice for compatibility [47,50].

Here, we employ an experimental design somewhat similar to [50], to eliminate the effect of mate quality: we compare the fitness of individuals that bred with their preferred partner with those that obtained, after having expressed their preference, the preferred partner of another individual. The main aim of this study is thus to quantify the benefits of mate choice that arise from partner compatibility, while circumventing confounding effects of variation in partner quality. The second aim of our study is to tease apart *indirect* compatibility advantages (compatibility of parental genes expressed in the offspring) from *direct* ones (parental phenotypic compatibility), using a model species in which these benefits of mate choice can be disentangled.

The zebra finch (*Taeniopygia guttata*) is a socially monogamous species with biparental care, in which partners mate for life [52]. In this species, female mate preferences are predominantly individual-specific (i.e., females show little consensus regarding which male is the most attractive) [15,53–57], suggesting that they may target genetic or behavioral compatibility. In captive and wild populations, high rates of embryo and offspring mortality are found, even in the absence of inbreeding [25,52,58–60]. Cross-fostering of freshly laid eggs (see [61,62] and S1 Text) showed that most of the variance in embryo mortality (before hatching) is explained by the identity of the genetic parents rather than the foster‐parents (based on *n* = 1,529 fertilized eggs, S1 Table), whereas most of the variance in offspring mortality (after hatching) is explained by foster-parent rather than genetic parent identity (*n* = 1106 offspring, S1 Table). Based on these results, we assume that, in zebra finches, embryo mortality primarily reflects genetic incompatibility (as in other species [63,64]), while offspring mortality primarily results from the behavior of the caring parents (here broadly referred to as “behavioral incompatibility”). Experimentally preventing mate choice should thus lead to an increase in embryo mortality if mate choice is targeting genetic compatibility, and to an increase in offspring mortality if mate choice is targeting behavioral compatibility. Alternatively, if individual-specific mate preferences only reflect indecision by the animal or measurement error [15], preventing mate choice would have no fitness consequences.

We studied 160 bachelor birds from a recently wild-derived population of zebra finches. Each individual could freely choose a partner from a group of 20 individuals of the opposite sex during a long, nonbreeding season. This setup reflects the natural situation in the sense that zebra finches are opportunistic breeders and do not reproduce if the environment is not suitable, but they still form life-long pair bonds irrespective of breeding opportunities. Furthermore, the species is gregarious, such that individuals have many potential partners to choose from. Pairs were identified by the occurrence of allopreening because we found that this best reflects mutual preferences rather than being the outcome of intra-sexual competition (see S2 Text). We hereafter focus on female preferences; however, because observed allopreening preferences were mainly reciprocal between females and males (see also S2 Text), any observed effects of the experimental treatment described below could be due to females, males, or both sexes not being able to breed with their (most) preferred partner. Females from these pairs were alternately assigned to one of two treatments: half of them were allowed to stay with their chosen partner, while the other half were force-paired with the chosen partner of another female from the same aviary. This ensured that, on average, individuals of both treatments were of the same quality, even if assortative pairing for quality had happened due to intra-sexual competition. All pairs were then placed in individual cages for a few months to enforce pair-bonding in the non-chosen pairs (force-pairing is effective in this species if assigned mates are co-housed in a cage for long enough, see “Methods”). After this period in separate cages, pairs were given the opportunity to breed for about five consecutive months (allowing about three successful broods) in communal aviaries, each containing three pairs from each treatment group. This entire procedure was repeated a second time with the same birds (i.e., free choice during a nonbreeding period, force pairing in cages, and breeding in communal aviaries). This was planned a priori to obtain repeated measurements on individuals under different pairing conditions with a large enough sample size to allow the detection of weak effects. For the second breeding period, two-thirds of the pairs from the first breeding period were broken up; individuals chose a new partner and were either assigned to the same or the other treatment. The other third of the pairs were allowed to keep their partner (chosen or non-chosen) from the first breeding period. This allowed us to better control for any effects of pair-bond duration in statistical models comparing chosen and non-chosen pairs, given that pair-bond formation in chosen pairs systematically started earlier (during the free choice period) than in non-chosen pairs (in cage). In total, we monitored behavior and reproductive success of 46 chosen pairs (C) and 38 non-chosen pairs (NC). Measures of reproductive success were based on paternity analyses that included dead embryos, dead chicks, and surviving offspring. Behavior was scored based on direct observations (285 h) and video recordings (1,424 h).

## Results

When released into communal breeding aviaries, each of which contained three chosen and three arranged pairs, the proportion of pairs that stayed together differed between treatment groups (C: 46 out of 50 pairs, NC: 38 out of 50 pairs; Fisher’s exact test *p* = 0.05). This suggests that birds that were force-paired with a partner they did not choose were more reluctant to breed together. However, this differential rate of divorce between the treatment groups is unlikely to induce a bias in our experimental results for the following reasons: (1) Individuals that divorced during one of the breeding periods did not appear to differ in intrinsic quality from individuals that stayed together, as judged from a comparison of reproductive success obtained in the other season when they did not divorce (relative fitness, mean ± standard error [SE]; divorced: 1.06 ± 0.13, *n* = 23; not divorced: 0.98 ± 0.08, *n* = 59; general mixed effect model accounting for treatment, birds matched for year, *p* = 0.58). (2) To induce a bias that is large enough to explain our results, the difference in intrinsic quality between divorcees and the remaining population would need to be unrealistically large (for further details, see simulation in S3 Text).

Only those pairs that remained together were considered for further analyses. Parameter estimates of traits for the two treatments (C versus NC) are given for each general and generalized linear mixed-effect model. Other relevant statistics, as well as the structure of the models, are provided in Table 1 (referred to as “T1-test #”).

**Table 1:** Treatment effect on breeding parameters and sexual behaviors. **The structure of each model (fixed and random effects) is given, as well as the mean estimates (±SE) of each treatment group.** Underlying data for each test can be found in S1 Data.

												Random effects				
#	Unit	*n*_total_	*n*_yes_	Dependent variable	*p*	*z*	C	±SE	NC	±SE	Fixed effects	FID	MID	PID	CID	Level
1	MID-yr	84		**Male relative fitness**	**0.03**	-2.24	1.16	0.11	0.80	0.12		55	54	70		
2	FID-yr	84		Female relative fitness	0.12	-1.59	1.09	0.11	0.84	0.12		55	54	70		
3	FID-yr	84		Number of eggs laid	0.56	0.58	13.5	1.12	14.4	1.18	**A**	55	54	70		Ass
4	Egg	1,172	320	*Disappeared egg*	*0*.*07*	1.83	12%	2%	19%	3%	**A**+**B**	55	54	69	222	Soc
5	Egg	707	167	Dead embryo	0.68	0.42	20%	4%	22%	4%	**A**+B	52	53	64	205	Gen
6	Offspring	594	245	**Dead offspring**	**0.03**	2.18	32%	5%	52%	7%	A+**C** ^**2**^	53	54	65	181	Soc
7	Offspring	421		Mass at 8 d old	0.51	-0.68	6.18	0.13	6.00	0.16	**A**+**D** ^**2**^	49	51	59	149	Soc
8	Clutch	216	39	**Infertile egg**	**0.01**	2.62	8%	3%	23%	5%	A+E	52	53	65		Ass
9	Clutch	245	44	Female EPY	0.39	-1.75	6%	3%	11%	5%	*A*+E+*K*	53				Gen
10	Clutch	209	55	Dumped egg	0.41	-0.82	26%	5%	21%	5%	**A**	54				Soc
11	Brood	181		*Brood size*	*0*.*07*	-1.82	2.59	0.21	2.03	0.23	A	53	54	65		Soc
12	Clutch	135		Clutch interval	0.57	-0.55	34.2	1.36	33.1	1.45	A+**F**+**G**			60		Soc
13	Day	2,081	532	Male attendance	0.24	-1.16	22%	2%	18%	3%	H+**I**	52	53	63	172	
14	Day	556	215	**Male attendance**	**0.04**	-2.11	42%	4%	30%	4%	H	52	53	63	170	
15	Courtship	2,503		**WP responsiveness**	**0.01**	-2.56	0.20	0.04	0.05	0.04	*A*+*J*+K+**L**+**M**	55	54	70		
16	Courtship	2,752		EP responsiveness	0.39	0.88	-0.53	0.02	-0.51	0.02	*A*+**J**+**K**+*L*+**M**	55	59	322		
17	Courtship	2,555	492	WP copulation	0.28	-1.09	20%	3%	16%	2%	**A**+**J**+K+**L**+M	55	54	70		
18	FID-yr	84	11	EP copulation	0.13	1.50	6%	4%	16%	6%	A+**N**	55				
19	MID-yr	84	15	EP copulation	0.64	-0.47	17%	6%	13%	6%	A+N		54			
20	MID-yr	84		WP courtship rate	0.46	0.74	0.48	0.04	0.52	0.05	**A**		54			
21	FID-yr	84		EP courtship rate	0.75	-0.33	0.36	0.08	0. 33	0.06	**A**+**K**	55				
22	MID-yr	84		**EP courtship rate**	**0.05**	2.03	0.20	0.07	0.34	0.07	**A**+**K**		54			
23	MID-yr	84	25	EPY	0.16	-1.39	32%	8%	17%	7%	*A*		54			

Column clarification and abbreviations:

**#**: test number given in the main text or supporting texts as T1-#.

**Unit**: unit of analysis for which sample sizes *n*_total_ are given. “MID-yr” (or “FID-yr”) stands for the combination of the male (or female) unique identity number and the year (2012 or 2013).

***n***_**yes**_: the number of yeses for binomial dependent variables.

**Dependent variables**: WP and EP stand for within and extra-pair, and EPY for extra-pair young. “Disappeared eggs”: eggs that were either buried in the nest material or broken before the end of incubation, or eggs that completely disappeared from the aviary; “Female EPY” is whether or not in a given clutch the female had any extra-pair young; “Brood size” is the number of offspring in a clutch that reached 8 d of age; “Male attendance” is whether or not the male attended its nest on a given daily nest check; “WP or EP copulation” are behaviorally successful copulations as opposed to unsuccessful courtships or copulation attempts.

***p*** and ***z***: *p*-value and *z*-value for the treatment.

**C** and **NC (±SE)**: parameter estimates for chosen and non-chosen pairs and their respective standard errors. For tests with binomial dependent variables, the average of the asymmetrical back-transformed standard errors is given.

**Fixed effect**: **A**: year; **B**: egg number in the laying sequence; **C**: hatching order (coded 1–8); **D**: the hatching order of the offspring among those that reached 8 d of age; **E**: clutch size; **F**: clutch number for the pair within a year (coded 1–6); **G**: brood size reached in the previous clutch of the same pair; **H**: number of offspring in the nest; **I**: a continuous variable counting up the days from the first day with offspring in that brood, reflecting the average age of the offspring; **J**: the number of days between the courtship and the day that is 3 d before the start of egg laying (with values ≥5 coded as 5), which means that 0 stands for the peak of fertility of that female; **K**: pair-bond duration (in days); **L**: the number of eggs the female laid in the last 5 d; **M**: the number of minutes after the light went on in the aviary on that day (artificial lights were set to turn on around 40 min before sunrise); **N**: number of courtships received, for females, or performed, for males. ^**2**^ indicates that a linear and a polynomial term were included using the function ‘poly’ in R. All fixed effects were centered.

**Random effects: FID**, **MID**, **PID,** and **CID**: the number of levels of random effects; female, male, pair, and clutch identity numbers (where applicable).

**Level**: level at which the analyses was performed, i.e., how the units were allocated to individuals. **Gen** stands for genetic parents, **Soc** for social parents, and **Ass** for assigned parents, which are genetic when parentage analysis was possible, and social parents when it was not (infertile and disappeared eggs).

Test #20, #21, and #22 are based on videotaped courtships only to obtain meaningful courtship rates as number of courtships per hour. Nevertheless, those tests were also done on all courtships observed (directly observed and videotaped), and gave similar results (see S5 Text).

Pair bond duration was accounted for in tests #9, #21, #22; i.e., when significant or indicating a trend (see Table 2) and for the test #15, #16, #17 for consistency with [53].

Bold characters in the table emphasize significance (*p* < 0.05); italic characters indicate trends (*p* < 0.10), tests of a priori hypotheses are underlined.

### Total Fitness: Mate Choice for Compatibility?

**Fig 2:**
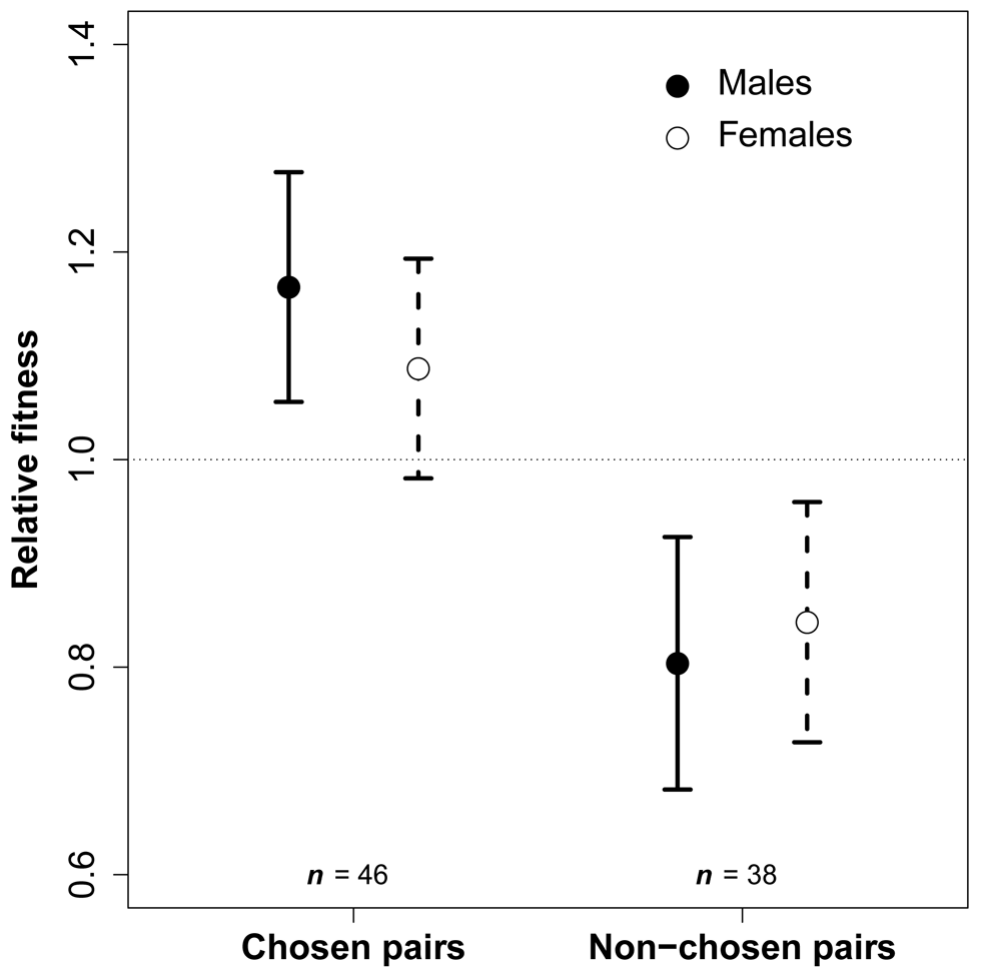
Relative fitness estimates (mean ± SE) of males (*n* = 84) and females (*n* = 84) from chosen and non-chosen pairs. Parameter estimates and SE were given by the general linear mixed-effect models T1-1 and T1-2. Underlying data for both tests can be found in S1 Data.

We calculated relative fitness of individuals as the total number of genetic offspring produced in a given breeding period that reached independence (35 d old), relative to the number produced in the same period by the other individuals in the same aviary. Males of chosen pairs had a 45% higher relative fitness than males of non-chosen pairs (C = 1.16, NC = 0.80, *p* = 0.03, *n* = 84 male breeding periods, see T1-1 for model details, Fig 2). Females of chosen pairs had a 30% higher relative fitness than those of non-chosen pairs, but the difference was not significant (C = 1.09, NC = 0.84, *p* = 0.12, *n* = 84, T1-2, Fig 2). The difference between the sexes was not significant (interaction between treatment and sex: *p* = 0.36) and resulted from extra-pair paternity (see below). Thus, on average, individuals from the chosen pairs had a 37% higher fitness. This difference in fitness was not due to differences in pair bond duration between the treatments groups, as this covariate did not correlate with fitness (non-significant trends against the expectation, Table 2: T2-1 and T2-2) and was therefore removed from the models T1-1 and T1-2.

**Table 2:** Effect of pair bond duration when included as a fixed effect in the models presented in Table 1. Underlying data for each test can be found in S1 Data.

					Pair bond duration effect		
#	Unit	*n*_total_	*n*_yes_	Dependent variable	Estimate	±SE	*p*
1	MID-yr	84		Male relative fitness	-0.09	0.15	0.53
2	FID-yr	84		Female relative fitness	-0.09	0.14	0.52
3	FID-yr	84		Number of eggs laid	-2.81	1.87	0.14
4	Egg	1,172	320	Disappeared egg	-0.03	0.42	0.94
5	Egg	707	167	Dead embryo	0.40	0.41	0.33
6	Offspring	594	245	Dead offspring	0.63	0.49	0.20
7	Offspring	421		Mass at 8 d old	0.10	0.23	0.70
8	Clutch	216	39	Infertile egg	0.69	0.69	0.31
9	Clutch	245	44	Female EPY	-1.70	0.99	*0*.*09*
10	Clutch	209	55	Dumped egg	0.36	0.53	0.50
11	Brood	181		Brood size	-0.46	0.44	0.11
12	Clutch	135		Clutch interval	3.58	3.17	0.25
13	Day	2,081	532	Male attendance	0.06	0.21	0.77
14	Day	556	215	Male attendance	-0.14	0.31	0.66
15	Courtship	2,503		WP responsiveness	-0.08	0.06	0.17
16	Courtship	2,752		EP responsiveness	-0.08	0.03	**<0.005**
17	Courtship	2,555	492	WP copulation	-0.06	0.26	0.81
18	FID-yr	84	11	EP copulation	-1.02	1.71	0.55
19	MID-yr	84	15	EP copulation	0.13	1.30	0.92
20	MID-yr	84		WP courtship rate	-0.03	0.08	0.69
21	FID-yr	84		EP courtship rate	-0.17	0.07	**0.01**
22	MID-yr	84		EP courtship rate	0.30	0.08	**<0.0003**
23	MID-yr	84	25	EPY	0.38	1.03	0.72

Estimates are presented on their original scale and reflect changes per 365 d (logit scale for binomial models, and square-root scale for models on courtship rates #20 to #22).

Bold characters in the table emphasize significance (*p* < 0.05); italic characters indicate trends (*p* < 0.10).

### Fitness Components: Genetic Versus Behavioral Compatibility?

The overall fitness difference observed was not due to differential investment in egg production by the females of the two treatment groups (total number of eggs laid: C = 13.5, NC = 14.4, *p* = 0.56, *n* = 84, T1-3). However, non-chosen pairs tended to have a higher proportion of disappeared or buried eggs (C = 12%, NC = 19%, *p* = 0.07, *n* = 1172 eggs laid, T1-4), and had significantly more clutches that contained infertile eggs (C = 8%, NC = 23%, *p* = 0.01, *n* = 216 clutches, T1-8).

**Fig 3:**
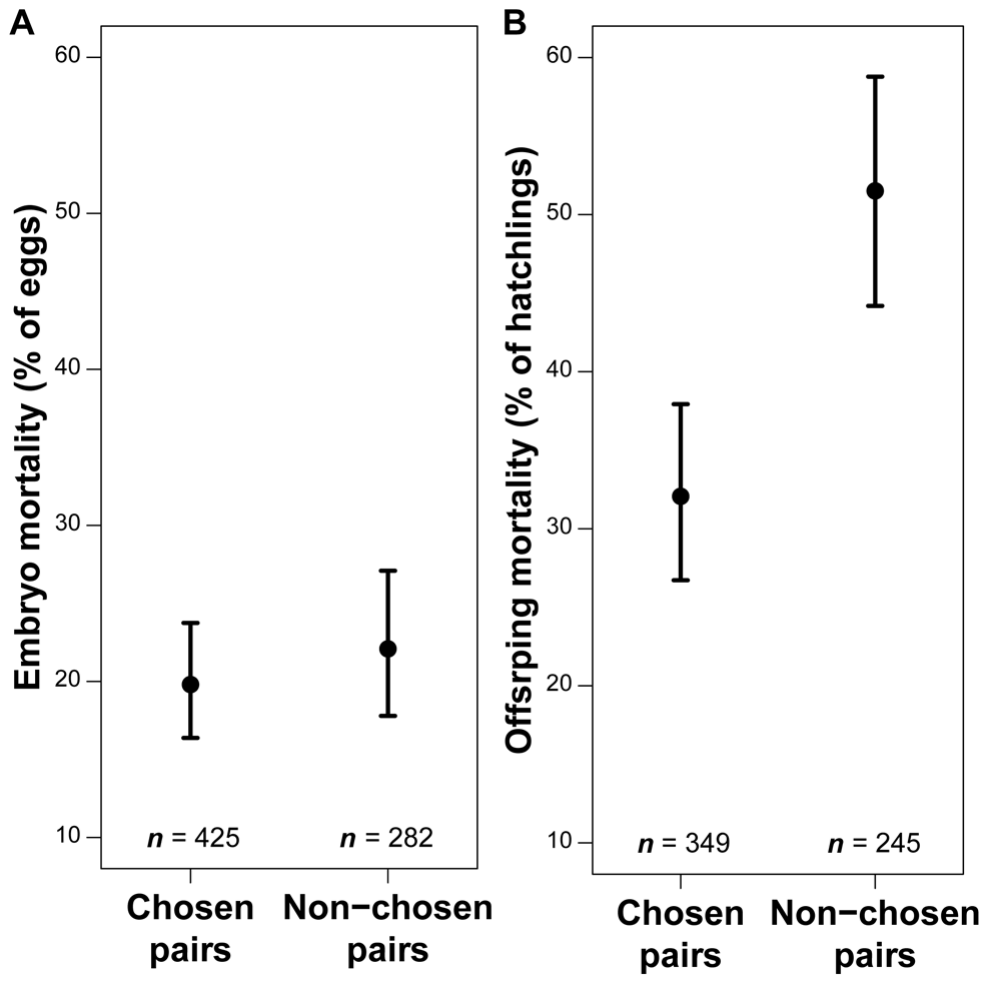
Embryo (A) and offspring (B) mortality rates (parameter estimates [mean ± SE]) in chosen and non-chosen pairs. Parameter estimates and SE were given by the generalized linear mixed-effect models T1-5 and T1-6 based on *n* = 707 fertilized eggs and *n* = 594 hatched eggs, respectively. Underlying data for both tests can be found in S1 Data.

To test the genetic incompatibility hypothesis, we compared the proportion of dead embryos between treatment groups, considering all fertilized and incubated eggs. We only included the genetic eggs of each pair, that is, we excluded all extra-pair young (9% of the eggs), but included eggs that were dumped into the nest of other pairs (13% of the genotyped eggs). Note that removing dumped eggs (potentially suffering higher rate of embryo mortality [65]) from the analysis did not change the conclusions. Furthermore, we only included eggs that were incubated without interruption, excluding those that were buried in the nest material before incubation was completed (based on daily nest checks). The rate of embryo mortality did not differ between chosen and non-chosen pairs (C = 20%, NC = 22%, *p* = 0.68, *n* = 707 fertilized eggs, T1-5, Fig 3A).

To test the behavioral compatibility hypothesis, we compared the proportion of dead offspring between treatment groups, considering all hatched eggs in a pair’s nest (including extra-pair offspring and hatchlings from dumped eggs). Offspring mortality was significantly higher when chicks were reared by non-chosen pairs (C = 32%, NC = 52%, *p* = 0.03, *n* = 594 hatched eggs, T1-6, Fig 3B). Pair bond duration did not influence this result (T2-6). The probability of survival may also decrease if the offspring is unrelated to one or both of the parents. To check this, we added the status of the offspring (within-pair versus extra-pair young, offspring from dumped versus not dumped egg) into model T1-6. We found that the treatment effect was still significant (*p* = 0.045), but offspring status was not (mortality of within-pair young = 38%, extra-pair young = 55%, *p* = 0.15; dumped = 40%, non-dumped = 39%, *p* = 0.91; underlying data can be found in S1 Data).

### Exploratory Analyses: Behavior of Chosen and Non-chosen Pairs

#### Within-pair courtship behavior

Males from both treatment groups courted their partner equally often (C = 0.48, NC = 0.52 courtships per hour, *p* = 0.46, *n* = 84 male breeding periods, T1-20). Yet, females were significantly less responsive to their partner during courtship if they were paired to a male they had not chosen themselves (C = 0.20, NC = 0.05, *p* = 0.01, *n* = 2,503 courtships, T1-15). Within-pair responsiveness was not affected by pair bond duration (T2-15). In non-chosen pairs, 16% of courtships led to copulation, which was not significantly lower than the rate of 20% in chosen pairs (*p* = 0.28, *n* = 2,555 courtships, T1-17).

#### Extra-pair courtship behavior and paternity

Females of both treatment groups were courted by extra-pair males equally often (C = 0.36, NC = 0.33 courtships per hour, *p* = 0.75, *n* = 84, T1-21), after controlling for the fact that females with longer pair bonds were courted significantly less (*p* = 0.01, T2-21). The response of females towards a courting extra-pair male was mostly negative, independent of the treatment group (C = -0.53, NC = -0.51, *p* = 0.39, *n* = 2,752 extra-pair courtships, T1-16), and significantly declined with pair bond duration (T2-16). Females of non-chosen pairs were only non-significantly more likely to engage in extra-pair copulations (C = 6%, NC = 16%, *p* = 0.13, *n* = 84 females, T1-18) and to have clutches that contained eggs sired by an extra-pair male (C = 6%, NC = 11%, *p* = 0.39, *n* = 245 clutches, T1-9), after controlling for the fact that females with longer pair bonds tended to have fewer extra-pair offspring (*p* = 0.09, T2-9). These tests for treatment effects on female promiscuity were somewhat inconclusive because relatively large estimated effects in the expected direction were still far from significance, indicating a shortage of statistical power. Males, for their part, had significantly higher extra-pair courtship rates when assigned to a partner (C = 0.20, NC = 0.34 courtships per hour, *p* = 0.05, *n* = 84, T1-22), after controlling for the fact that males from pairs with longer pair bonds engaged more in extra-pair courtships (*p* < 0.001, T2-22). Males of both treatment groups were equally likely to obtain an extra-pair copulation (C = 17%, NC = 13%, *p* = 0.64, *n* = 84 courting extra-pair males, T1-19), and males of chosen pairs were non-significantly more successful in siring at least one extra-pair young (C = 32%, NC = 17%, *p* = 0.16, *n* = 84, T1-23).

#### Nest attendance

Male nest attendance showed a pronounced peak when eggs hatched (S1 Fig). Most of the offspring that died (58%), died within 24–48 h after hatching (i.e., maximally seen once alive on daily nest checks). Thus, we considered nest attendance during the entire offspring rearing period, and also specifically on those days during which one or more offspring hatched in a given nest. Males of non-chosen pairs attended their nest significantly less than those of chosen pairs during the days of hatching (C = 42%, NC = 30%, *p* = 0.04, *n* = 556 d of hatching, T1-14), but not during the entire offspring rearing period (C = 22%, NC = 18%, *p* = 0.24, *n* = 2,081 d with nestlings, T1-13).

#### “Pair harmony.”

To test whether pairs showing more behavioral compatibility before breeding will have higher reproductive success [23], we recorded the occurrence of affiliative versus agonistic behavior, the propensity of individuals to follow their partner, and the distance and synchrony in activity between the members of a pair. These measures, as well as courtship rates and female responsiveness to courtships, were entered into a principal component analysis (PCA). Chosen and non-chosen pairs differed significantly in their PC1 score (C = 0.24, NC = -0.29, *p* = 0.01, *n* = 84, S3 Table), with individuals of chosen pairs mainly staying closer together and behaving more synchronously than those of non-chosen pairs (S2 and S3 Tables). PC1 only weakly predicted pair fitness, though the trend was in the expected direction (r = 0.18, *n* = 84, *p* = 0.11, S4 Table).

The choice treatment did not have any effect on egg dumping (T1-10), brood size (T1-11), chick mass at day 8 (T1-7), and the interval between clutches (T1-12; see S4 Text).

## Discussion

Many studies have attempted to quantify the benefits of mate choice [2–7,35–46,48–50,66,67], but only a few have quantified the fitness benefits of mate choice for compatibility while excluding quality benefits (see e.g. [46] and [50]) (Fig 1). Our experimental design allowed us to circumvent the potentially confounding effect of mate quality by comparing pairs of individuals that chose each other with pairs that were composed of random individuals who did not choose each other, but had both been chosen by another individual. Pairs that formed through free mate choice had a 37% higher fitness than pairs that were “forced” experimentally (Fig 2). This suggests that it is unlikely that the between-individual disagreement about mate attractiveness simply reflects indecision or measurement error. Our results suggest instead that individual-specific mate preferences lead to significant fitness consequences.

Our study system, furthermore, allowed us to disentangle *direct* (behavioral) benefits of mate choice from *indirect* (genetic) benefits (Fig 1). Chosen pairs, compared to arranged ones, had a 38% lower rate of offspring mortality (Fig 3B). Under the assumption that offspring mortality systematically depends on parental behavior, this result supports the hypothesis of mate choice for behavioral compatibility. Ideally, our experiment should be repeated while cross-fostering eggs to exclude confounding factors. Indeed, our conclusions depend on the generalizability of the results from our previous study (S1 Text). The finding that offspring mortality after hatching primarily depends on the rearing parents and not on the genetic parents (S1 Table) can likely be generalized from our previous cross-fostering experiment to this study; in both studies, many offspring apparently died from starvation, and an offspring that is not fed will die irrespective of its genetic quality. Finally, chosen and arranged pairs had an equal rate of embryo mortality (Fig 3A). Given that embryo mortality primarily depends on the genetic parents and less on the incubating parents (S1 Table), this result argues against the hypothesis of mate choice for genetic compatibility. At least, our results suggest that individuals did not select a partner with whom they would have minimized the rate of embryo mortality.

Several earlier experimental studies favored the genetic compatibility hypothesis based on the observation that offspring from “free-choice” pairs had a higher viability than those from “forced” pairs [35–37,40,43,46,66]. However, in these experiments females were forced to mate with random males from the population or with non-preferred males, some of which may have been of lower absolute quality (but see [46]). Hence, the previously observed effects on offspring viability may be explained by differences in both genetic quality and compatibility. In general, mate choice for genetic compatibility may not easily evolve, because it requires that the incompatibility-causing loci are tightly linked (e.g., via pleiotropy) to a detectable phenotype and to a mechanism ensuring the appropriate assortative or disassortative preference [68]. At least in zebra finches, such a complex adaptation that would allow them to minimize embryo mortality by choosing a genetically compatible partner, does not seem to exist (this study). Similarly, inbreeding avoidance is absent in this species when birds can only judge genetic similarity per se [61] (although it does take place when siblings are familiar with each other [69]).

Our results are consistent with the hypothesis that behavioral compatibility between the pair members leads to benefits of mate choice. This could come about through different mechanisms: the emerging behaviors of a pair in terms of coordination or complementarity [23,24,27–29], and/or the individual-specific stimulation of a partner’s sensory system leading to a greater investment in reproduction [32–34]. Currently it is unclear which of these factors leads to the observed variation in parental care compatibility, and it is also unclear to what extent there is a genetic basis for this variation in compatibility. In the following, we discuss our exploratory analyses on fitness components and behaviors of “free-choice” and “forced” pairs, to provide testable ideas about how such behavioral compatibility benefits could arise.

We found that non-chosen pairs (1) more often had clutches with infertile eggs, (2) had more offspring dying at an early stage (presumably from starvation), and (3) tended to have more eggs that disappeared (presumably due to poorer care and nest defense). These effects on components of fitness may be due to differences in the behavior of chosen and non-chosen pairs.

The most prominent behavioral differences were that (a) females with assigned partners responded less positively to within-pair courtship and they tended to copulate less frequently with their partner, and (b) males with assigned partners showed poorer nest attendance during the egg hatching period. The females’ reduced tendency to participate in within-pair courtship and copulation when in a “forced” pair may explain the higher incidence of infertile eggs. Indeed, in a previous experiment in which continuous video recording allowed us to witness about 80% of all copulations over a 4-mo period (partly reported in [53]) we found that the probability of laying an infertile egg declined significantly with the number of copulations witnessed during the 10 d prior to egg laying (*p* = 0.04, *n* = 376 eggs laid by 31 females, estimates: 27% infertile at 0 copulations versus 15% infertile at the median of 5 copulations; underlying data can be found in S1 Data). Alternatively, apparently infertile eggs may in fact represent cases of very early embryo mortality. This seems unlikely because egg fertility scores in zebra finches were tightly linked to the number of sperm that reached the egg [70]. Likewise, the lower nest attendance during hatching by males in non-chosen pairs could indicate a reduced motivation to care for the young or defend the nest when in a forced partnership, leading to greater offspring mortality and egg loss. Consequently, the results of these exploratory analyses further support the behavioral compatibility hypothesis.

If males and females in “forced” pairs indeed invest less in reproduction (copulation or care), as our results suggest, the question remains why. Reduced investment by members of “forced” pairs could be a long-term effect of a single stressful event (trauma), namely the loss of the chosen partner (an event that could also happen in the wild due to predation [71]). This explanation seems unlikely, however, because fitness was affected by the treatment per se and not by the number of partner losses experienced by an individual (see scheme in “Methods”) when both factors were fitted within one model (males: treatment *p* = 0.02, number of mate losses *p* = 0.63; females: treatment *p* = 0.06, number of mate losses *p* = 0.35). Alternatively, being forced to breed with a non-preferred partner (unlikely to occur in the wild) might cause chronic stress. Being chronically stressed when paired to a specific partner A but not when paired to partner B would be part of the “phenotypic incompatibility” phenomenon.

Our score of “pair harmony,” which was based on affiliative and sexual behaviors, as well as behavioral synchrony and the tendency to reunite, did not significantly correlate with pair fitness. A study on zebra finches in the wild reported that behavioral synchrony was associated with brood size [25], but further experimental work suggested that variation in synchrony might have been the consequence and not the cause of variation in reproductive success [72].

Evidence supporting the idea that pair coordination is important mainly comes from studies showing an increase in breeding success with pair bond duration ([27–29,73,74] but see [75]). We specifically designed our experiment to create variation in pair-bond duration (pairs stayed together for one or two breeding periods). However, this covariate did not have an effect on any of the fitness components (mostly showing non-significant trends opposite to expectation, Table 2) and was therefore removed from most final models. This suggests that behavioral compatibility (with synergistic effects on fitness) did not increase with pair bond duration.

The only traits that were affected by pair-bond duration were those related to extra-pair behavior (Table 2): females responded less positively to extra-pair courtships and received fewer extra-pair courtships with increasing pair-bond duration. In contrast, male courtship rate towards extra-pair females *increased* with pair-bond duration. In other words, it seems that females decreased and males increased their promiscuous behavior.

It has been suggested that individuals choose each other based on their respective personality, which would determine their behavioral compatibility [22]. Individuals that show similar behavioral types, or similar plasticity (and therefore predictability), could be better at negotiating or coordinating their actions, and could therefore have reduced conflicts over parental care and higher reproductive success ([26,76,77] but see [78]). So far, besides observational studies [76,79,80], only two experiments (both conducted on zebra finches) aimed at testing this hypothesis, and none of them found consistent evidence for pair combination effects on rearing success, based on any of the personality traits measured [26,78]. We did not measure any personality traits of individuals prior to the experiment, because we did not have clear a priori predictions about the advantages of being behaviorally similar. Instead, we scored the synchrony of activities during breeding, but this did not differ between treatment groups (see S4 Text).

Although an effect of lack of coordination between pair members cannot be excluded, our exploratory analyses suggest a reduced investment or commitment in individuals of “forced” pairs (lower female within-pair responsiveness, higher male extra-pair courtship rate, lower male nest attendance). Previous experimental work on zebra finches shows that the amount of male singing activity can affect egg quality [33]. More generally, courtship and other affiliative behaviors, which may occur more frequently in chosen pairs, may affect the level of reciprocal stimulation [32,81,82]. Earlier studies that favored the genetic compatibility hypothesis cannot rule out that the treatment (chosen versus non-chosen pairs) affected maternal investment (e.g., egg quality) with potential effects on offspring viability [35–37,43,46]. Artificial insemination would be needed to experimentally demonstrate that higher offspring viability arises from genetic compatibility and not from maternal (e.g., egg nutrients) or paternal effects (e.g., sperm allocation) following greater stimulation by a preferred partner (see e.g., [5]).

If forced pairs reduced their investment in breeding together, as our analyses suggest, the question remains whether this behavior is adaptive. Reduced investment in current reproduction could be adaptive, if it saves resources for future reproduction with a better (preferred) partner. However, this explanation seems unlikely for a species such as the zebra finch, because life-long monogamy largely precludes breeding with a different partner in the future [52]. Moreover, in a follow-up experiment consisting of a third breeding season where all individuals could freely choose their mate, individuals could not compensate for the lower fitness previously obtained with a non-chosen partner (see S6 Text). Therefore, the reduced investment in breeding by members of non-chosen pairs could be maladaptive, either because this never occurs under natural conditions (because individuals are never forced to mate or breed with a particular partner), or because some constraints limit the adaptive behavioral flexibility of the animals.

To conclude, chosen pairs had significantly higher fitness than forced pairs, apparently due to behavioral rather than genetic compatibility effects. The mechanisms behind such behavioral compatibility, in terms of willingness or ability to cooperate with certain individuals and in terms of coordination between partners need further study, in particular in the context of offspring provisioning. In humans, some studies suggest that individuals are more satisfied, more committed, and less likely to engage in domestic violence, when involved in a love-based rather than an arranged marriage ([83,84], but see [85]). The challenge there is also to find out whether stable and happy marriages result from motivation to cooperate (and to identify what stimulates such feelings, see [86–89]), or from congruence in terms of partners’ intrinsic behavioral types [90].

## Methods

The study was approved by the Animal Care and Ethics Committee of the Max Planck Institute for Ornithology.

### Design

**Fig 4:**
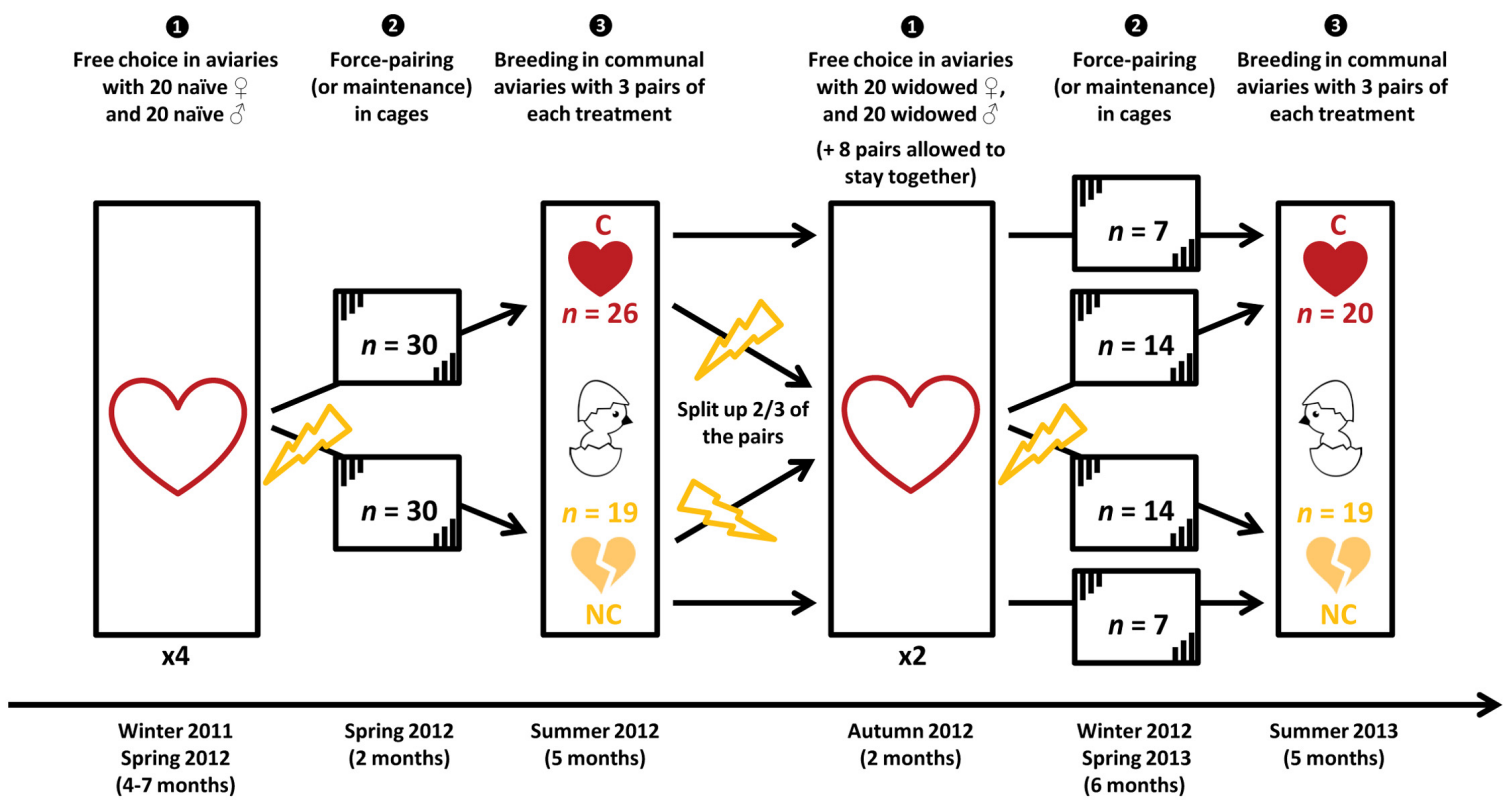
Experimental design and timeline. Chosen pairs (C, *n* = 46, filled red hearts) resulted from free choice. Non-chosen pairs (NC, *n* = 38, broken yellow hearts) resulted from force-pairing (by being put together in a cage; experimental stage 2) between individuals that expressed a choice (during experimental stage 1), but who were separated from their initial chosen partner (event symbolized by yellow lightning). Fitness of all pairs was measured during experimental stage 3. The follow-up study (S6 Text) took place in spring 2014. All experimental birds had hatched in the summer of 2011.

A scheme of the design with its timeline is depicted in Fig 4. All experimental birds hatched in the summer of 2011 in large semi-outdoor aviaries. The origin of the birds (population #4 in [91]), and rearing and housing conditions have been described in detail elsewhere [60]. This population has been derived from the wild only about ten generations ago [91]. Shortly after independence (age 45 d), individuals were put into eight mixed-sex peer-groups of ten males and ten females. When birds reached sexual maturity (100 d old) they were color-banded, and peer-groups were joined two by two (yielding four groups, each allowing 20 possible pairs to form). Sixty-six pairs were identified during ad libitum observations in the winter of 2011–2012. Mid-April 2012, half of the identified pairs were randomly assigned to the treatment group NC (in which all birds are assigned the partner of someone else: “non-chosen”), while the remaining pairs went to the treatment group C (in which birds are allowed to stay with their chosen partner). In order to induce pair formation in the randomly created pairs of the NC group, these pairs were put into individual cages for a period of two months and were allowed to lay one clutch. Pairs from the C group also went to such cages and were allowed to lay one clutch in order to standardize all experiences apart from the re-pairing. On the 21^st^ of May, three pairs of each treatment group (chosen randomly but excluding the initial chosen partners of individuals of non-chosen pairs) were put into a breeding aviary (10 replicates, 60 pairs in total). Both members of each pair had been previously color-banded on both legs with one random color out of six (dark blue, light blue, black, yellow, orange, white), so that a pair would be unmistakably identifiable in its aviary. Forty-five pairs (26 C, 19 NC) did not divorce and were considered for the analyses. After one week of intensive focal pair observations, we introduced nest material, and checked nests daily until 21 August, when the experiment was stopped and newly laid eggs were replaced by dummy eggs, but pairs were still allowed to raise all offspring from eggs laid before that.

In October 2012, once all offspring had reached independence, we assigned treatment groups for the second breeding season. First, we randomly selected eight pairs from each treatment group (among the 26 C and 19 NC that were available) that were allowed to stay with their partner throughout the second season. In this way, we could better separate the effects of choice treatment from the potential effects of pair-bond duration. These 16 pairs and all other adults (remaining C, NC, and previously divorced birds) were put into one of two big flocks, to allow a second round of choosing a partner. Each group contained 20 widowed males (i.e., their former breeding partner was in the other group), 20 widowed females, and eight established pairs. As a result, each widowed female could choose a new partner among a set of 20 new males, which never included her previous breeding partner (but for half of the females from non-chosen pairs it did include again the initially chosen mate because this could not be avoided). In December 2012, after pair identification and random assignment to treatment group (without regard to previous treatment), pairs were put into cages for six months and allowed to lay two clutches. The longer period of force-pairing in cages resulted in a lower divorce rate compared to the previous season (only one pair of each treatment divorced). On 21 May 2013, pairs were put again into breeding aviaries and allowed to breed as in the previous year. Of 52 pairs identified in the winter groups, 42 (21 of each treatment group, across seven aviaries) contributed to the second breeding period (12 birds died accidentally because food dispensers were blocked for 2 d in early March 2013). The design itself and the accidental food shortage may have led to selection of the highest quality individuals. Although it did not induce bias (selection was independent of treatment group), it can result in an underestimation of the real fitness benefits of mate choice. Furthermore, one member of a pair died for unknown reasons (and its partner was removed) within the first week of each breeding period (1 C in 2012 and 1 NC in 2013), and these two pairs were excluded from the analyses.

### Breeding Monitoring

Each aviary contained 7 nest boxes. Every morning, all nests were checked, the individual(s) attending the nest identified, and the fate of each egg and each offspring noted. Unhatched eggs were opened when neglected by the parents (for instance, after offspring had fledged) and embryos were collected for parentage analysis (using 11 microsatellite markers, following [92] and [93]). For the same purpose, small (~10 μl) blood samples were taken from 8–10 d old offspring, or tissue samples if they died earlier. Of 1,434 eggs laid by all birds including divorcees, 28% (*n* = 402) could not be assigned through parentage analysis, and were assigned to the social pair that attended the nest. These eggs included apparently infertile eggs (5.6%, *n* = 80), and eggs that were buried in the nest and did not develop (typically after a nest take-over by another pair) or disappeared (presumably they broke and were eaten by the birds) (21.6%, *n* = 310), as well as eight dead embryos and four dead hatchlings that yielded bad DNA samples. Relative fitness of an individual was calculated as the total number of genetic offspring produced in a given breeding period that reached independence (age 35 d) divided by the average number of offspring produced by all same-sex individuals of the same aviary that did not divorce.

### Behavioral Observations

Each aviary was equipped with a dome camera set to record different aviary positions during each day of the week. During 3 d, we filmed an artificial tree, on which 69% of all courtships took place (calculated from direct observations, described below). For one day, we recorded each of the two sets of nest boxes, and for 2 d, a set of perches on which individuals often allopreen. We analyzed the first hour of each day, when copulations are most frequent [53]. In all pairs considered for the analyses (those that did not divorce), we recorded 1,942 within-pair (WP) and 2,999 extra-pair (EP) courtships (in the latter, a divorced female or male may have been the extra-pair partner). For each courtship, we scored female responsiveness as follows: threat or aggression toward the male (−1), flying away (−0.5), mixed or ambiguous signs (0), courtship hopping and beak wiping (+0.5), and copulation solicitation (+1), and noted whether it led to a successful copulation.

We also conducted direct observations, following a protocol inspired by studies on cockatiels, *Nymphicus hollandicus* [21,23,94]. Observations were carried out both in the pre-breeding period (first week after release into aviaries before nesting material was added) and during the entire breeding period, to test whether pairs with greater behavioral compatibility before breeding (as in [23]), or during breeding activities, would have greater reproductive success. The observer stood behind a one-way glass window (built into each aviary door) and carried out focal-pair watches by monitoring a pair for 3 min. During these watches we observed 613 WP and 800 EP courtships. We noted their location and whether they led to a successful copulation. For a subset of 561 WP and 782 EP courtships, we also scored female responsiveness, as described above. During focal-pair watches, we also recorded whether within-pair allopreening or aggression occurred during the 3 min period (“yes” or “no”). Every 30 s, we recorded the distance between the partners and their activity. Distance was averaged for each 3 min watch. Activities were split into nine categories: feeding, cleaning, nesting or parental behavior (nest building or attendance, and feeding of fledglings), sleeping, sitting, involved in aggression, involved in courtship, flying, and “other.” We defined pair synchrony as the sum of the observations in which both partners engaged in the same activity (range 0–6). For each pair member, we also recorded all occurrences of an individual flying away from or back towards (<50 cm) its partner (e.g., female flying away: Faway, male flying back: Mback). From those counts, we calculated the tendency of the pair to reunite: (*Σ Fback* + *Σ Mback*) / (*Σ Faway* + *Σ Maway*), and a mate guarding index: (*Σ Faway* − *Σ Fback*) − (*Σ Maway* − *Σ Mback*). The latter is positive in case of male mate guarding, and negative for female mate guarding. The six pairs in an aviary were watched successively in a randomized predetermined order, and the time of observation of each aviary was randomized over the course of each day (i.e., from sunrise to sunset). In 2012, pairs were watched 9–13 times (median = 11) in the pre-breeding period, and 37–39 times (median = 38) during the breeding period. In 2013, 16–21 focal watches (median = 21) per pair were performed during pre-breeding, and 68–70 (median = 69) during breeding. For each pair, all measures were averaged for all focal watches separately for the pre-breeding and breeding period, because these periods were analyzed separately as planned a priori (see Results and S4 Text). Male courtship rates (WP and EP courtships per hour) and best linear unbiased predictors (BLUPs, i.e., random effect estimates) of female responsiveness (to WP and EP courtships) were also calculated (see S5 Text) for both periods and included in a principal component analysis (PCA). All observations were done blind to the treatment of the birds.

### Data Analyses

All statistical tests were conducted in R [95]. General and generalized mixed-effect models were performed with the “lmer” and “glmer” function of the lme4 package [96] and the PCAs with the “principal” function of the psych package [97]. All fixed effects were chosen a priori by considering (a) their biological relevance (e.g., hatching order when looking at offspring mortality), (b) their mathematical relevance (e.g., clutch size when looking at the presence of infertile eggs in a clutch), (c) the experimental design (e.g., year), and (d) consistency with previously published models (e.g., how to model the fertile period when looking at female responsiveness). *P*-values for general mixed effect models (lmer) were obtained from model comparison (with and without the explanatory variable) with the function anova in R; *p*-values for generalized mixed effect models (glmer) were taken from the model output (calculated from *z*-values).

## Supporting Information

S1 Data Excel spreadsheet containing, in separate sheets, the underlying numerical data for each test presented in Tables 1 and 2, S1 Table, part A of S3 and S4 Tables, part B of S3 and S4 Tables, S6 Text Males and Females, S1 Fig, and for the analysis presented in the discussion (the relationship between egg fertility and the number of observed copulations). (XLSX)

S1 Fig Proportion of daily nest checks in which the female, the male, or both pair members simultaneously attended their nest while having an active clutch or a brood. The values for joint nest attendance are included in the values for females and males. Day 1 is the start of incubation. In zebra finches, incubation lasts on average 12 d, and offspring fledge 15–20 d after hatching.
(TIF)

S1 Table Causes of embryo and offspring mortality in cross-fostered eggs shown as variance component estimates. The analysis is described in S1 Text. Results are from a domesticated population (#18 in [45]).
(PDF)

S2 Table Loadings of behavioral variables on PC1 for the pre-breeding (A), and the breeding period (B). (PDF)

S3 Table Results of linear models investigating the effect of the treatment on PC1 scores and on each variable included in the PCA separately, for the pre-breeding (A) and the breeding period (B). For all tests, *n* = 84 pairs.
(PDF)

S4 Table Results of linear models investigating the effect of PC1, and each variable included in the PCA separately, on the relative fitness achieved by the pairs. **Analyses for the pre-breeding (A) and the breeding period (B) are shown.** For all tests, *n* = 84 pairs.
(PDF)

S1 Text Causes of offspring and embryo mortality in zebra finches: egg cross-fostering experiments. (PDF)

S2 Text Pilot study on the assessment of mating preferences. (PDF)

S3 Text Analysis and discussion of the differential rates of divorce between chosen and non-chosen pairs. (PDF)

S4 Text Treatment effects on other fitness-related traits and behaviors. (PDF)

S5 Text Details on behavioral variables used in the PCA. (PDF)

S6 Text Follow-up experiment: opportunity to compensate later in life. (PDF)
