# Supplementary figures and images for: Fitness Benefits of Mate Choice for Compatibility in a Socially Monogamous Species
Source: PLoS Biol. 2015 Sep 14;13(9):e1002248. doi: 10.1371/journal.pbio.1002248 (PMC4569426; doi:10.1371/journal.pbio.1002248)

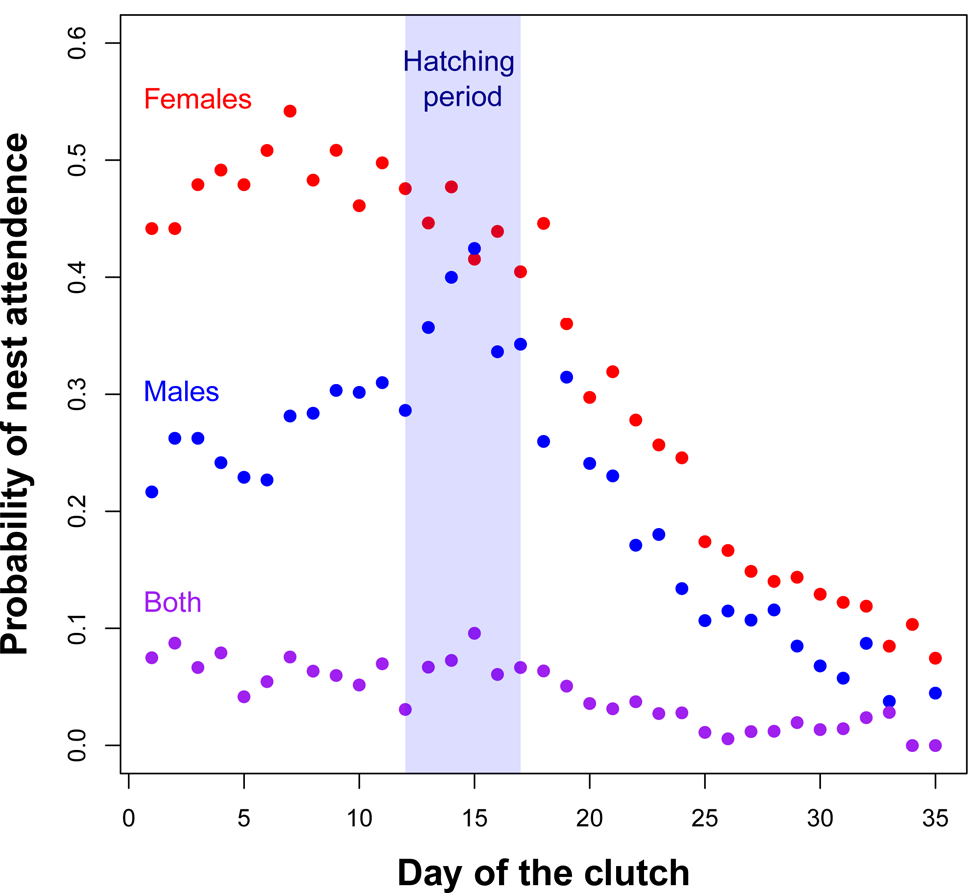

Supplement: S1 Fig — The values for joint nest attendance are included in the values for females and males. Day 1 is the start of incubation. In zebra finches, incubation lasts on average 12 d, and offspring fledge 15–20 d after hatching. (TIF) [file pbio.1002248.s002.tif]
