# Supplementary material for: Fitness Benefits of Mate Choice for Compatibility in a Socially Monogamous Species
Source: PLoS Biol. 2015 Sep 14;13(9):e1002248. doi: 10.1371/journal.pbio.1002248 (PMC4569426; doi:10.1371/journal.pbio.1002248)
Supplement: S1 Table — The analysis is described in S1 Text. Results are from a domesticated population (#18 in [45]). (PDF) [file pbio.1002248.s003.pdf]

**S1 Table. Causes of embryo and offspring mortality in cross-fostered eggs shown as variance component estimates (Varcomp).**

| Dependent variable | Unit | $n_{total}$ | $n_{died}$ | Mortality | *n* genetic pairs | *n* foster pairs | Varcomp genetic pair ID | Varcomp foster pair ID | % genetic | % foster |
|---|---|---|---|---|---|---|---|---|---|---|
| Dead embryo | Egg | 1529 | 423 | 28% | 280 | 260 | 0.6569 | 0.2896 | 69.4% | 30.6% |
| Dead offspring | Hatchling | 1106 | 283 | 26% | 243 | 252 | 0.2079 | 0.9966 | 17.3% | 82.7% |
