# Supplementary material for: Fitness Benefits of Mate Choice for Compatibility in a Socially Monogamous Species
Source: PLoS Biol. 2015 Sep 14;13(9):e1002248. doi: 10.1371/journal.pbio.1002248 (PMC4569426; doi:10.1371/journal.pbio.1002248)
Supplement: S2 Table — (PDF) [file pbio.1002248.s004.pdf]

S2 Table. Loadings of behavioral variables on PC1 for the pre-breeding (A), and the breeding period (B).

A. pre-breeding period

Variable	Loading
Mean distance	-0.74
EP courtship rate*	-0.48
Female EP responsiveness†	-0.20
Female WP responsiveness †	-0.17
Male aggression	-0.09
Female aggression	-0.03
Female allopreening	0.28
WP courtship rate*	0.39
Male allopreening	0.41
Mate guarding	0.48
Synchrony‡	0.57
Proportion of flight back§	0.61

B. breeding period

Variable	Loading
Mean distance	-0.86
Female WP responsiveness †	-0.28
Female EP responsiveness†	-0.09
EP courtship rate*	0.04
Female aggression	0.11
Male aggression	0.15
Proportion of flight back§	0.22
Mate guarding	0.36
Female allopreening	0.66
Male allopreening	0.70
WP courtship rate*	0.74
Synchrony‡	0.79

*Courtship rates (within-pair (WP), and extra-pair (EP)) were calculated from both videotaped courtships and courtships observed directly, see S5 Text.

†BLUPs of female responsiveness in WP or EP courtships, obtained from linear mixed-effect models with the same structure as T1-15 and T1-16, see S5 Text.

‡Synchrony was z-transformed within year, see S5 Text.

§Pair tendency of reuniting, see *Methods.*
