# Supplementary material for: Fitness Benefits of Mate Choice for Compatibility in a Socially Monogamous Species
Source: PLoS Biol. 2015 Sep 14;13(9):e1002248. doi: 10.1371/journal.pbio.1002248 (PMC4569426; doi:10.1371/journal.pbio.1002248)
Supplement: S3 Table — For all tests, n = 84 pairs. (PDF) [file pbio.1002248.s005.pdf]

S3 Table. Results of linear models investigating the effect of the treatment on PC1 scores and on each variable included in the PCA separately, for the pre-breeding (A) and the breeding period (B).

A. pre-breeding period

Dependent Variable	*p*	*z*	C	±SEM	NC	±SEM
**PC1**	**0.01**	-2.50	0.24	0.14	-0.29	0.21
**Mean distance**	**<0.00**	3.20	40	3.0	54	3.3
**EP courtship rate**	**<0.05**	2.02	-0.17	0.13	0.21	0.14
Female EP responsiveness	0.91	0.12	0.00	0.01	0.00	0.01
Female WP responsiveness	0.30	-1.04	0.02	0.02	-0.02	0.03
Male aggression	0.28	1.08	0.01	0.00	0.02	0.01
Female aggression	0.75	-0.31	0.01	0.00	0.01	0.00
Female allopreening	0.46	-0.75	0.14	0.02	0.12	0.02
WP courtship rate	0.97	0.05	0.00	0.12	0.00	0.13
Male allopreening	0.16	-1.41	0.25	0.02	0.20	0.02
Mate guarding	0.97	-0.04	0.65	0.13	0.65	0.14
**Synchrony**	**0.02**	-2.35	0.23	0.14	-0.27	0.16
Proportion of flight back	0.87	-0.16	0.89	0.01	0.88	0.02

B. breeding period

Dependent Variable	*p*	*z*	C	±SEM	NC	±SEM
PC1	0.91	-0.12	0.01	0.15	-0.01	0.22
Mean distance	0.34	0.97	120	4.1	126	4.5
Female WP responsiveness	0.14	-1.50	0.03	0.03	-0.03	0.03
Female EP responsiveness	0.35	0.94	0.00	0.01	0.00	0.01
EP courtship rate	0.41	0.83	-0.07	0.13	0.09	0.14
Female aggression	0.22	1.24	0.00	0.00	0.00	0.00
Male aggression	0.31	1.03	0.00	0.00	0.00	0.00
Proportion of flight back	0.99	0.01	0.82	0.01	0.82	0.01
Mate guarding	0.76	-0.31	0.15	0.06	0.13	0.06
Female allopreening	0.22	-1.24	0.06	0.01	0.04	0.01
Male allopreening	0.74	-0.33	0.08	0.01	0.08	0.01
WP courtship rate	0.21	1.27	-0.11	0.13	0.13	0.14
Synchrony	0.98	0.03	0.00	0.15	0.00	0.16

More information on the dependent variables is given in the footnotes of S2 Table. Bold characters emphasize significance.
