# Supplementary material for: Fitness Benefits of Mate Choice for Compatibility in a Socially Monogamous Species
Source: PLoS Biol. 2015 Sep 14;13(9):e1002248. doi: 10.1371/journal.pbio.1002248 (PMC4569426; doi:10.1371/journal.pbio.1002248)
Supplement: S4 Table — Analyses for the pre-breeding (A) and the breeding period (B) are shown. For all tests, n = 84 pairs. (PDF) [file pbio.1002248.s006.pdf]

S4 Table. Results of linear models investigating the effect of PC1, and each variable included in the PCA separately, on the relative fitness achieved by the pairs. Analyses for the pre-breeding (A) and the breeding period (B) are shown.

A. pre-breeding period

explanatory variable	*p*	*z*	*r*
PC1	*0.11*	1.62	0.18
Mean distance	*0.06*	-1.88	0.20
EP courtship rate	0.64	-0.47	0.05
Female EP responsiveness	0.90	-0.13	0.01
Female WP responsiveness	0.18	1.37	0.15
Male aggression	*0.06*	1.92	0.21
Female aggression	0.36	0.92	0.10
Female allopreening	0.60	0.53	0.06
WP courtship rate	0.32	1.00	0.11
Male allopreening	0.63	0.49	0.05
Mate guarding	0.46	0.74	0.08
Synchrony	0.84	0.20	0.02
Proportion of flight back	*0.07*	1.85	0.20

B. breeding period

explanatory variable	*p*	*z*	β
PC1	0.93	-0.12	0.01
Mean distance	0.43	-0.80	-0.10
Female WP responsiveness	0.59	0.54	0.05
**Female EP responsiveness**	**0.01**	-2.83	-0.27
EP courtship rate	0.18	-1.35	-0.13
**Female aggression**	**0.02**	-2.38	-0.23
Male aggression	*0.10*	-1.65	-0.16
Proportion of flight back	0.88	0.15	0.01
Mate guarding	0.85	-0.19	-0.02
Female allopreening	0.87	-0.17	-0.02
Male allopreening	0.87	-0.17	-0.02
WP courtship rate	0.81	-0.24	-0.03
Synchrony	0.45	0.76	0.09

More information on the explanatory variables is given in the footnotes of S2 Table. The number of days the pair was actively breeding (incubating eggs or rearing offspring) during the breeding period (B) was including as covariate for all tests. If this covariate is not included, male and female allopreening, as well as synchrony, within-pair courtship rate, and the overall PC1, are significantly negatively related to pair fitness because individuals that are not engaged in breeding activities (e.g. because their brood failed) spent more time on non-reproductive activities. Hence, this highly significant covariate of fitness is always controlled for. The coefficient of correlation *r*, or the standardized regression coefficient β (equivalent to the correlation coefficient (and identical if the covariate is removed)), are given to estimate the correlations between the relative fitness of pairs and the explanatory variables. β was obtained with the function 'lm.beta' from the package QuantPsyc [103], in R [95].

Bold characters emphasize significance, italic characters indicate trends.

Supplemental references

103. Fletcher TD (2012) QuantPsyc: quantitative psychology tools. R package version 1.5. http://CRAN.R-project.org/package=QuantPsyc.
