# Supplementary material for: Fitness Benefits of Mate Choice for Compatibility in a Socially Monogamous Species
Source: PLoS Biol. 2015 Sep 14;13(9):e1002248. doi: 10.1371/journal.pbio.1002248 (PMC4569426; doi:10.1371/journal.pbio.1002248)
Supplement: S1 Text — (PDF) [file pbio.1002248.s007.pdf]

S1 Text. Causes of offspring and embryo mortality in zebra finches: egg cross-fostering experiments.

To assess to what extent embryo and offspring mortality depend on the parents that produce the eggs (genetic effects) versus the parents that care for the eggs (rearing effect), cross-fostering eggs individually within the experiment would have been necessary. However, such cross-fostering would have invalidated our measurements of fitness for the two treatment groups by reducing the between-clutch variance in hatching success. Therefore, and also to maximize power, we used data from a more extensive cross-fostering experiment that had been carried out in our domesticated population over the course of two generations (F1 and F2), as previously described [61,62,98]. Eggs were cross-fostered individually within 24 h after being laid such that a pair's clutch of e.g. five eggs would get spread among five foster nests, and each foster clutch of e.g. five eggs would be composed of eggs from five different genetic pairs. From a total of 2,128 eggs that were cross-fostered between 2004 and 2006, 1,529 eggs had been incubated through the whole incubation period and were genotyped for parentage [65]. The remaining eggs were either infertile, dried out or broken. The 1,529 eggs originated from 280 different pairs of genetic parents and were cared for by 260 different pairs of foster parents. Of those eggs, 423 (28%) died before hatching, leading to 1,106 offspring, 283 (26%) of which died during offspring rearing (i.e. before reaching independence at 35 days of age). For each egg and offspring we coded mortality as yes or no, and extracted the variance component estimates for the two random effects "genetic pair identity" and "foster pair identity" from mixed effect models with binomial error structure using the lme4 package [96] from R [95] and without specifying any fixed effects besides the intercept (see S1 Table). The predicted rate of embryo mortality varied between pairs from 18% to 54%; while for chick mortality, it varied between 16% and 40%.

S1 Table shows that in our zebra finch population embryo mortality is primarily a trait of the genetic pair, while offspring mortality primarily depends on the rearing parents.

Supplemental references

98. Forstmeier W (2005) Quantitative genetics and behavioural correlates of digit ratio in the zebra finch. Proc R Soc B 272: 2641-2649.
