# Supplementary material for: Fitness Benefits of Mate Choice for Compatibility in a Socially Monogamous Species
Source: PLoS Biol. 2015 Sep 14;13(9):e1002248. doi: 10.1371/journal.pbio.1002248 (PMC4569426; doi:10.1371/journal.pbio.1002248)
Supplement: S2 Text — (PDF) [file pbio.1002248.s008.pdf]

S2 Text. Pilot study on the assessment of mating preferences.

A pilot study was performed in 2011 to confirm (a) that mutual allopreening strongly predicts which pairs will end up breeding together [99], and (b) that allopreening is a more accurate assessment of mating preferences than realized mating patterns per se. Two flocks of 20 unpaired males and 20 unpaired females were first observed to identify allopreening pairs, and were then given access to nest material and nest boxes. Twenty-nine out of 32 pairs (91%) that ended up breeding in the nest boxes, had been previously identified based on allopreening. The three other pairs resulted from changes owing to mortality (1 pair) and intrasexual competition (2 pairs). In these latter cases, two females (and two males respectively) preferred the same male (the same female, respectively), and intra-sexual aggression decided about realized pairings. As we never observed individuals aggressively preventing allopreening, we concluded that the assessment of mating preferences was best evaluated by the occurrence of allopreening.

Supplemental references

99. Silcox AP, Evans SM (1982) Factors affecting the formation and maintenance of pair bonds in the zebra finch, Taeniopygia guttata. Animal Behaviour 30: 1237-1243.
