# Supplementary material for: Fitness Benefits of Mate Choice for Compatibility in a Socially Monogamous Species
Source: PLoS Biol. 2015 Sep 14;13(9):e1002248. doi: 10.1371/journal.pbio.1002248 (PMC4569426; doi:10.1371/journal.pbio.1002248)
Supplement: S3 Text — (PDF) [file pbio.1002248.s009.pdf]

S3 Text. Analysis and discussion of the differential rates of divorce between chosen and non-chosen pairs.

Each treatment group initially consisted of 50 pairs, of which 4 dropped out in the C group (leaving *n* = 46) and 12 in the NC group (leaving *n* = 38). By how much would dropout pairs have to differ from non-dropouts in order to induce a given difference between the remaining 46 C pairs and the 38 NC pairs? We addressed this by running a simple simulation on the data of relative fitness in males (which is defined to have a mean of 1). We assigned the same value x of hypothetical relative fitness to each dropout male (4 C and 12 NC) and added these 16 fitness values to the list of observed relative fitness values for the remaining 46 C and 38 NC males. We then progressively increased the value of x until the mean of the joint 50 NC males equaled the mean of the 50 C males (i.e. the null hypothesis), which was the case for x=2.6. Hence, dropout males need to have a relative fitness of 2.6 in order to induce a bias in the remaining population of the magnitude we observe (Fig. 2). This seems highly unlikely, given that only 1 out of 84 males (46+38) ever achieved such a high relative fitness.

In order to explain all observed effects as byproducts of dropout, the divorcing pair members (both the deserting individuals and the ones left behind) would need to be of higher intrinsic quality, show a higher responsiveness in within-pair courtships, a higher male nest attentiveness, and a lower rate of infertility. This seems unlikely under the "better option hypothesis", which represents the conceptual framework that has received most attention and support [100-102]. It predicts that members that are mismatched for quality are the most likely to divorce. The high-quality individual would abandon the low-quality partner, and would repair with a higher-quality individual who again would leave behind a lower-quality partner. Such an event would always take out four individuals of both higher and lower quality from our pool of birds and therefore limit the scope for divorce to create an overall bias.

We therefore argue that pairs that divorced might either have been mismatched for intrinsic quality (which should not induce a bias), or have been particularly behaviorally incompatible (which would lead to an underestimation of the treatment effects in the remaining pairs, making our conclusions conservative but less precise).

Supplemental references

100. Choudhury S (1995) Divorce in birds - a review of the hypotheses. Anim Behav 50: 413-429.
101. Ens BJ, Safriel UN, Harris MP (1993) Divorce in the long-lived and monogamous oystercatcher, Haematopus ostralegus - incompatibility or choosing the better option? Anim Behav 45: 1199-1217.
102. Otter K, Ratcliffe L (1996) Female initiated divorce in a monogamous songbird: abandoning mates for males of higher quality. Proc R Soc B 263: 351-355.
