# Supplementary material for: Fitness Benefits of Mate Choice for Compatibility in a Socially Monogamous Species
Source: PLoS Biol. 2015 Sep 14;13(9):e1002248. doi: 10.1371/journal.pbio.1002248 (PMC4569426; doi:10.1371/journal.pbio.1002248)
Supplement: S4 Text — (PDF) [file pbio.1002248.s010.pdf]

S4 Text. Treatment effects on other fitness-related traits and behaviors.

We tested whether egg-dumping rates were higher in nests of non-chosen pairs, for example because they defended them less well against conspecific parasitism. However, both types of pairs were equally likely to attend clutches that contained dumped eggs (C = 26%, NC = 21%, $p = 0.41$, $n = 209$ clutches, T1-10).

Although “forced” pairs started with a similar clutch size, and showed a similar rate of embryo mortality (see main text), they had slightly more disappeared or buried eggs and dead offspring (see main text). In sum, this resulted in a tendency for non-chosen pairs to have a lower brood size (number of 8 days old offspring: C = 2.59, NC = 2.03, $p = 0.07$, $n = 181$ broods, T1-11).

If non-chosen pairs are behaviorally less compatible or less motivated to raise the brood (see main text), they might end up with offspring of lower body mass. However, offspring from “free choice” and “forced” pairs did not differ in mass at day 8 post-hatch (C = 6.18 g, NC = 6.00 g, $p = 0.51$, $n = 421$, T1-7), perhaps because “forced” pairs tended to have fewer offspring (see above).

We also tested whether chosen pairs had a higher breeding rate, that is, a shorter interval between clutches, for example if the male from free-choice pairs would be more likely to take care of the fledglings such that the female could start incubating a new clutch. However, chosen and non-chosen pairs took equally long before starting another clutch (C = 34.2 d, NC = 33.1 d, $p = 0.57$, $n = 135$ intervals between clutches, T1-12).

Finally, in our experiment, in addition to observing behavioral compatibility of pairs before breeding [23], observations were also carried out during the entire breeding period (every other day in 2012 and every day in 2013), to test whether coordination during breeding activities *per se* would correlate with reproductive success. These behavioral variables were combined in a principal component analysis (see *Methods* and the next section). The PC1 scores obtained for each pair during breeding did not differ between the treatments (Table B in S3 Table) and did not explain any variation in fitness (Table B in S4 Table).
