# Supplementary material for: Fitness Benefits of Mate Choice for Compatibility in a Socially Monogamous Species
Source: PLoS Biol. 2015 Sep 14;13(9):e1002248. doi: 10.1371/journal.pbio.1002248 (PMC4569426; doi:10.1371/journal.pbio.1002248)
Supplement: S5 Text — (PDF) [file pbio.1002248.s011.pdf]

S5 Text. Details on behavioral variables used in the PCA.

Courtship rates were first calculated for video and direct observations separately, and then combined (after standardization, see below). For the videos, the number of courtships (within-pair, WP, and extra-pair, EP) by a male observed from a certain camera position was divided by the number of hours of video watched from this position. The courtship rates obtained for a male were then summed across all camera positions. This avoids creating a bias for individuals with a preference for a certain courting location. Courtship rates calculated from direct observations were simply the number of courtships (WP and EP) observed in the entire aviary for a male, divided by the number of hours the aviary was monitored (focal pair watches). To reach normality, all courtship rates were square-root transformed.

Pair synchrony (as defined in *Methods*) was significantly lower and courtship rates were higher in the second year, possibly due to different observers or to slightly different protocols. Hence, synchrony scores and courtship rates were standardized (z-transformed) within years. Because of this complexity – introduced to give a fair weight to each observation – it is not possible to back-transform model estimates into number of courtships per hour. This is why analyses concerning courtship rates presented in the main text (and in S2 Table) were on videotaped courtships only. All tests were also conducted on courtship rates calculated as described above for the PCA (i.e. including direct observations), and the results obtained were similar.

Female responsiveness to male courtship depended on time of day and on the duration of the pair bond (see [53] and Table 1). Therefore, the random effect estimates of each female (BLUPs) were obtained for each observation period (pre-breeding and breeding period) from linear mixed-effect models consistently structured as in [53] and Table 1 (e.g. T1-15 and T1-16). Within and extra-pair responsiveness BLUPs of each female-season were used in the PCAs.
