# Supplementary material for: Fitness Benefits of Mate Choice for Compatibility in a Socially Monogamous Species
Source: PLoS Biol. 2015 Sep 14;13(9):e1002248. doi: 10.1371/journal.pbio.1002248 (PMC4569426; doi:10.1371/journal.pbio.1002248)
Supplement: S6 Text — (PDF) [file pbio.1002248.s012.pdf]

S6 Text. Follow-up experiment: opportunity to compensate later in life.

We designed a follow-up experiment to examine whether individuals of the non-chosen pairs were adaptively reducing their brood size by saving energy for potential future reproduction with a chosen partner. In order to give every individual a maximum of free choice and an opportunity to form a new pair bond, all existing pairs were split up after the second breeding period, by putting them in large unisex groups for six months. Then, all adults whose previous breeding partner was still alive (n = 35 pairs, 17 C and 18 NC) were placed together in a large breeding aviary for one month. We investigated how many pairs of each treatment re-united with their former breeding partner after the artificial 6-months separation. Birds had unique combinations of color-bands, and pairs were identified by the occurrence of affiliative and nesting behavior. All observations were done blind to previous pair bonds and treatments. Eggs were replaced by dummy eggs and put in an incubator for 5 days after which developing embryos (n = 114) were collected for parentage analysis (this time using 19 microsatellite markers). Relative fitness was calculated as the number of eggs an individual had sired (male) or laid (female) divided by the average number of eggs produced by individuals of the same sex. Each individual was given a score as follows: 0 for individuals that had bred with a chosen partner in both breeding seasons (C-C), 1 for individuals that had bred twice with a non-chosen partner (NC-NC), and 0.5 for individuals that had undergone one of each treatment (C-NC or NC-C). One female with score 0 died after two weeks in the experiment, without having formed a pair bond.

In total, 26 heterosexual pairs, one female-female pair, one polygynous trio and one polyandrous trio showed strong affiliative behaviors. Three chosen and two non-chosen pairs reunited out of 5 chosen and 6 non-chosen pairs that had been allowed to breed together for both experimental breeding seasons. In addition, 4 pairs reunited out of 11 chosen pairs from the second experimental breeding season, as well as 1 pair out of 12 non-chosen pairs. Thus, although chosen pairs were more likely to reunite, the effect was not significant (Mantel-Haenszel chi-squared test with continuity correction, p = 0.17; controlling for pair bond duration (1 or 2 years)). Interestingly, two females reunited with their chosen partner of the second breeding period with whom they had not been allowed to breed (out of 8 possible such cases), and were involved in one of the two trios. Finally, one female bonded with her chosen breeding partner of the first season (out of 6 possible such cases). The 18 other pairs were new combinations of partners.

We found that relative fitness under free choice in the follow-up experiment was not affected by previous treatments. The regression slopes of relative fitness over treatment score (coded as indicated above) were not greater than zero as would have been expected under the hypothesis of compensation later in life (female slope = -0.19, p = 0.59; male slope = -0.19, p = 0.66). These slopes correspond to relative fitness values of C-C = 1.08 and NC-NC=0.88 for females and C-C = 1.10 and NC-NC = 0.90 for males.

In conclusion, in a third breeding period where free mate choice was fully allowed individuals did not compensate for the lower fitness previously obtained with a non-chosen partner. This suggests that the reduction in investment by forced pairs is non-adaptive in this species.
